# *Phlogiellus bundokalbo* spider venom: cytotoxic fractions against human lung adenocarcinoma (A549) cells

**DOI:** 10.1590/1678-9199-JVATITD-2019-0104

**Published:** 2020-08-03

**Authors:** Anna Beatriz R. Mayor, Leonardo A. Guevarra, Myla R. Santiago-Bautista, Librado A. Santiago

**Affiliations:** 1 The Graduate School, University of Santo Tomas, Manila, Philippines.; 2 Research Center for Natural and Applied Sciences, University of Santo Tomas, Manila, Philippines.; 3 Department of Biochemistry, Faculty of Pharmacy, University of Santo Tomas, Manila, Philippines.

**Keywords:** Phlogiellus bundokalbo, Spider venom, Human lung adenocarcinoma, Philippine spiders, Cytotoxicity

## Abstract

**Background:**

Spider venom is a potential source of pharmacologically important compounds. Previous studies on spider venoms reported the presence of bioactive molecules that possess cell-modulating activities. Despite these claims, sparse scientific evidence is available on the cytotoxic mechanisms in relation to the components of the spider venom. In this study, we aimed to determine the cytotoxic fractions of the spider venom extracted from *Phlogiellus bundokalbo* and to ascertain the possible mechanism of toxicity towards human lung adenocarcinoma (A549) cells.

**Methods:**

Spider venom was extracted by electrostimulation. Components of the extracted venom were separated by reversed-phase high performance liquid chromatography (RP-HPLC) using a linear gradient of 0.1% trifluoroacetic acid (TFA) in water and 0.1% TFA in 95% acetonitrile (ACN). Cytotoxic activity was evaluated by the MTT assay. Apoptotic or necrotic cell death was assessed by microscopic evaluation in the presence of Hoechst 33342 and Annexin V, Alexa Fluor^TM^ 488 conjugate fluorescent stains, and caspase activation assay. Phospholipase A_2_ (PLA_2_) activity of the cytotoxic fractions were also measured.

**Results:**

We observed and isolated six fractions from the venom of *P. bundokalbo* collected from Aurora, Zamboanga del Sur. Four of these fractions displayed cytotoxic activities. Fractions AT5-1, AT5-3, and AT5-4 were found to be apoptotic while AT5-6, the least polar among the cytotoxic components, was observed to induce necrosis. PLA_2_ activity also showed cytotoxicity in all fractions but presented no relationship between specific activity of PLA_2_ and cytotoxicity.

**Conclusion:**

The venom of *P. bundokalbo* spider, an endemic tarantula species in the Philippines, contains components that were able to induce either apoptosis or necrosis in A549 cells.

## Background

Spider venoms possess a wide variety of pharmacologically active components such as low-molecular-weight compounds including organic toxins, peptides, enzymes and other neurotoxins which may have potential medical applications [1]. Previous studies reported the activity of spider venom components in preventing stroke-induced brain damage, Alzheimer’s disease, epilepsy, cardiovascular problems and cancer [2]. These activities are primarily attributed to either ion-channel-modulating activity, cell-surface-receptor-binding activity or catalytic activities of these components, which trigger various cell-signaling pathways that modulate cell functions [3, 4]. 

Among the numerous life-threatening diseases in the Philippines, lung cancer ranks second in fatalities among men and third among women [5]. New technologies and targeted treatments were introduced to decrease the number of deaths. However, such methods depend on the patient’s response for their effectiveness, and sometimes cause harmful side effects such as excessive exposure to radiation and development of drug resistance that present an additional threat to the patients. In this context, other potential therapeutic strategies should be studied in order to provide additional options and further insights into the prognosis and treatment of lung cancer.

Anti-cancer-related studies of spider venom components suggested different mechanisms in relation to their cytotoxic activities. Spider venom serves as an antagonist of glutamate receptors, induces apoptosis, necrosis, and anoikis, and modulates ion channels (calcium, potassium, and sodium). It also contains pore-forming peptides, and enzymes such as hyaluronidases and phospholipases, which influence cancer cell death [6]. However, sparse information is available about the cytotoxic mechanism of Philippine spider venom towards cancer cells. Therefore, this study aimed to identify the cytotoxic fractions of the spider venom from *Phlogiellus bundokalbo* and ascertain the mechanism of toxicity in A549 human lung carcinoma cells.

## Methods

### Venom Collection and Fractionation

The spiders were collected from Aurora, Zamboanga del Sur by a team from the Mindanao State University - Iligan Institute of Technology (MSU-IIT), and were identified at the University of the Philippines Los Baños Museum of Natural History as *Phlogiellus bundokalbo.* The spiders were kept and reared at the Department of Biochemistry, School of Pharmacy, University of Santo Tomas, for venom collection. The venom from these spiders was obtained by electrical stimulation based on a previously described method [7]. After collection, the spider venom samples were lyophilized and the dry weight of each sample was determined using an analytical balance. 

Components of the venom were separated by reverse-phase high performance liquid chromatography (RP-HPLC) [8]. One hundred microliters *P. bundokalbo* crude venom (AT005) was injected, and fractionated using a Waters E2695 HPLC system (Massachusetts, United States of America) and Agilent Eclipse Plus C18 column (5um, 4.6 x 150mm). Venom fractionation was performed using 0.1% trifluoroacetic acid (TFA) in water (Solvent A) and 0.1% TFA in 90% acetonitrile (Solvent B). Separation employed a linear gradient of 5-95% solvent B done in 100 minutes at a flow rate of 1 mL/min. The elution of the venom components was monitored at 215 nm and fractions were collected according to the appearance of peaks. Fractions were labeled and lyophilized, and the dry weight of each sample was determined using an analytical balance.

### MTT Cytotoxicity Assay

Prior to the assay, lyophilized crude spider venom and its fractions were weighed on an analytical balance. Different concentrations (10, 50, 100, and 200 µg/mL) of the samples were prepared by dissolving them in a specified volume of cell culture medium.

In a 96-well sterile microplate, human lung adenocarcinoma (A549) cells (ATCC^(^ CCL-185) at 1x10^4^ cells/well density were treated with different concentrations of crude spider venom and its fractions for 48 hours at 37°C with 5% CO_2_ supply and 98% humidity. Similar concentrations (10, 50, 100, and 200 µg/mL) of Cytosplat (Cisplatin) were prepared and served as the positive control, whereas the culture medium was used as the negative control. The cells were washed with PBS and 50(L of sodium 3’-[1-phenylamino-carbonyl)-3,4-tetrazolium]-bis (4-methoxy-6-nitro) benzene-sulfonic acid hydrate (MTT) at 1 mg/mL concentration was added to each well. The cells were incubated for 4 hours before the addition of 100 µL dimethyl sulfoxide and absorbance was read at 570nm using Multiskan GO Microplate Spectrophotometer (Thermo Scientific, USA) [9]. Average triplicate readings and blanks were utilized to determine the inhibition rate. This latter step was performed in four trials.

### Morphological Characterization of Spider Venom-Treated A549 Cells

A549 cells (5x10^5^ cells/mL) were treated with different concentrations (10, 50, 100, and 200 µg/mL) of crude spider venom and its fractions (10, 50, 100, and 200 µg/mL) for 48 hours at 37°C with 5% CO_2_ supply and 98% humidity. Cisplatin (10 µg/mL) was used as the positive control, whereas the culture medium was employed as the negative control. After incubation, the cells were washed with PBS. Dye solution containing Hoechst 33342 and Annexin V, Alexa Fluor^TM^ 488 conjugate dyes were loaded to each well, and the cells were observed under EVOS FL Cell Imaging System (Life Technologies, Singapore) after a 10-minute incubation period in the dark at 37°C [10]. 

### Caspase 3/7 Assay

Activation of caspase by the spider venom and its fractions was determined using a Promega Caspase-Glo® 3/7 Assay kit. The kit contained 100 mL Caspase-Glo® 3/7 buffer and a bottle of Caspase-Glo® 3/7 lyophilized substrate, which contains tetrapeptide sequence DEVD, in a reagent optimized for caspase activity, luciferase activity and cell lysis. The assay buffer was added to the substrate to prepare the Caspase-Glo® 3/7 reagent.

The A549 cells (1x10^6^ cells/mL) were treated with different concentrations of crude spider venom (10, 50, 100, and 200 µg/mL), venom fractions (10, 50, 100, and 200 µg/mL) and standard cisplatin (10 µg/mL) for 48 hours. After incubation, the cells were added to a similar volume of Caspase-Glo® 3/7 reagent and incubated for 1 hour. Luminescence was measured utilizing a GloMax® Discoverer Multimode Microplate Reader (Promega) [11]. Addition of a single Caspase-Glo 3/7 reagent to the treated cells resulted in cell lysis, followed by caspase cleavage of the substrate and generation of luminescent signal by luciferase (Ultra-Glo^TM^ Recombinant Luciferase) proportional to the amount of caspase activity present. Luminescence was measured for three trials with triplicate readings.

### Determination of Phospholipase A_2_ Activity

Phospholipase A_2_ activity was evaluated through a colorimetric assay using L-α-lecithin as the substrate. In a microcentrifuge tube, reaction buffer was prepared consisting of 100 μL buffer (500mM Tris-HCl + 10mM CaCl_2_, pH 8.5), 250 μL 1% lecithin, and 70 μL 1.5% deoxycholate. The tube was incubated at 37°C for 5 mins. Then in a 96-well microplate, 10 μL of venom fractions and standard bee venom was supplemented with 40 μL reaction buffer. The mixture was incubated at 37°C for 5 mins. Next, 180 μL 25% ether in ethanol, 15 μL 2M hydroxylamine, and 15 μL 14% NaOH were added. The plate was incubated for 20 mins at room temperature, after which 30 μL of 3N HCl and 30 μL of 10% FeCl_3_ were added to the solution. Absorbance was read immediately at 570nm using a Multiskan GO Microplate Spectrophotometer (Thermo Scientific, USA). A standard curve using different concentrations of lecithin and CaCl_2_ was utilized to compute the activity of PLA_2_.

### Statistical Analysis

The results were expressed as mean ± error of mean (SEM), and statistical comparisons were made using two-way analysis of variance (ANOVA) followed by Tukey’s and Dunnet’s Post Hoc tests, and paired Student’s *t* test to compare means. A value of p < 0.05 indicated significance. Median inhibitory concentration of each venom sample and fraction were determined via the software GraphPad Prism 6.

## Results

### Partial Purification of Spider Venom Components

**Figure 1.**
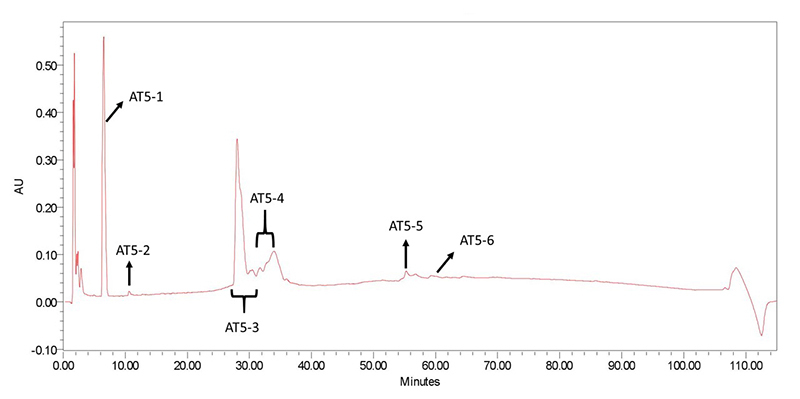
Chromatogram of *P. bundokalbo* crude spider venom using C18 column: absorbance was measured at 215nm.

Several peaks, starting from 6.00 min up to 68.00 minutes after sample injection, were observed (Figure 1). A single resolved peak at 6.51 minutes, followed by several other peaks starting from 23 to 68 minutes. The observed peaks were collected and labeled accordingly as shown in Figure 1. 

### **Cytotoxicity of *P. bundokalbo* Spider Venom and Its Fractions towards A549 Cells**

**Figure 2.**
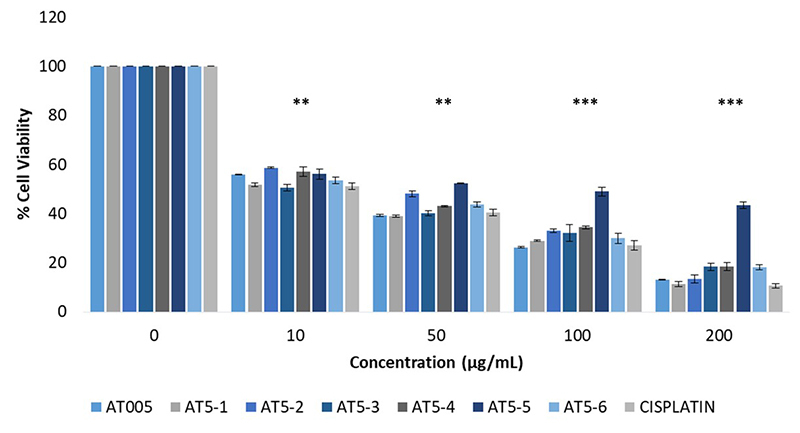
Concentration-dependent inhibition of A549 cells by *P. bundokalbo* venom fractions after 48-hour incubation period. Averaged data from three trials with triplicate readings are shown. ***Statistically different effect compared to the negative control, statistical difference between concentrations, and statistical difference between samples. **Statistically different effect compared to the negative control, and statistical difference between samples.

Figure 2 demonstrates the concentration-dependent inhibition of A549 cells by the AT005 (IC_50_ = 25.12 µg/mL), its fractions and cisplatin (IC_50_ = 14.50 µg/mL). Interestingly, variation was observed in activity between the collected fractions. Among the fractions, AT5-3 had the highest inhibitory activity with an IC_50_ value of 13.18 µg/mL, followed by AT5-1 (IC_50_ = 14.55 µg/mL), AT5-6 (IC_50_ = 17.34 µg/mL), AT5-4 (IC_50_ = 21.52 µg/mL), AT5-2 (IC_50_ = 24.89 µg/mL), and AT5-5 (IC_50_ = 58.48 µg/mL).

### Changes in the Morphological Features of A549 Cells after Treatment of Spider Venom

**Figure 3.**
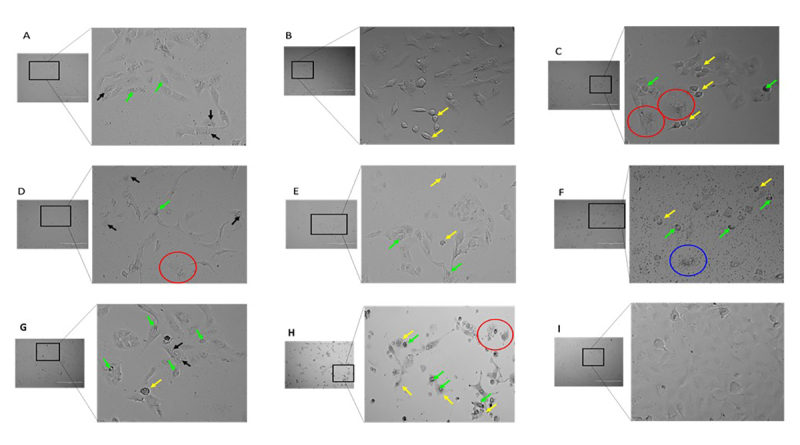
Photographs of A549 cells at 48 hours post-treatment with 200 µg/mL concentration of different AT005 spider venom fractions: **(A)** AT5-1, **(B)** AT5-2, **(C)** AT5-3, **(D)** AT5-4, **(E)** AT5-5, and **(F)** AT5-6, **(G)** Crude AT005, **(H)** 10 µg/mL cisplatin, and **(I)** untreated A549 cells. Arrow indicates different cytological features of A549 cells: black = membrane blebbing; yellow = cell shrinkage; green = nuclear condensation. Red circle = apoptotic cells; blue circle = lysed cell. Magnification: 40x; scale bars represent 40 µm.

**Figure 4.**
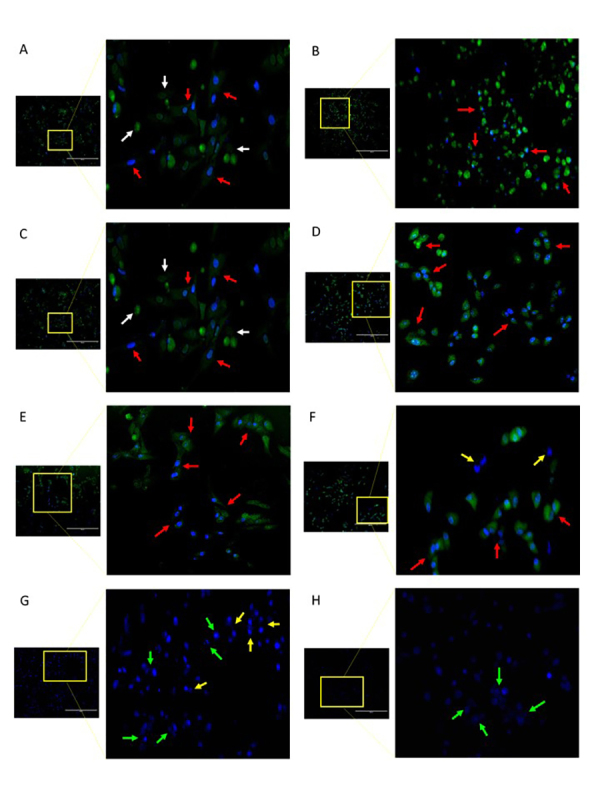
Photographs of A549 cells at 48 hours post-treatment with 200 µg/mL concentration of **(A)** crude spider venom, **(B)** cisplatin, and different spider venom fractions: **(C)** AT5-1, **(D)** AT5-2, **(E)** AT5-3, **(F)** AT5-4, **(G)** AT5-5, and **(H)** AT5-6. Arrow indicates different cytological features of A549 cells: red = apoptotic cells; white = apoptotic bodies; yellow = mitotic cells; green = compromised nuclear membrane. Magnification: 40x; scale bars represent 40 µm.

Reduction of cellular adhesion, cell condensation and membrane-blebbing were found in A549 cells treated with high concentrations of fraction AT5-3 (200 µg/mL) (Figure 3). These morphological characteristics were more pronounced in A549 cells treated with 10 µg/mL of cisplatin. On the other hand, membrane disintegration was observed in A549 cells treated with fraction AT5-6 (200 µg/mL). In addition, A549-treated cells presented fluorescence of bright green and blue coloration (Figure 4). Annexin V, Alexa Fluor 488^TM^ conjugate is a green fluorescent dye that specifically binds to phosphatidylserine (PS) that are released by the cells to their surface during apoptosis. Meanwhile, the blue color observed in cells was due to the binding of Hoechst 33342 fluorescent dye to the condensed DNA of the A549-treated cells. 

### **Caspase 3/7 Activation of *P. bundokalbo* Spider Venom**

**Figure 5.**
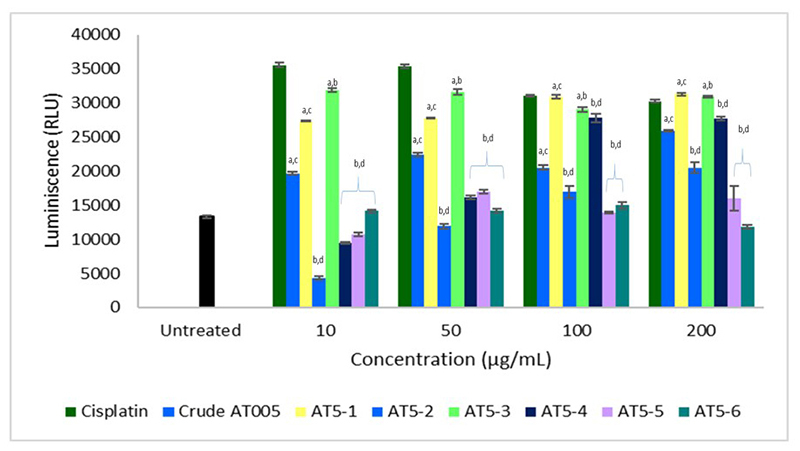
Caspase 3/7 activation of *Phlogiellus bundokalbo* crude spider venom and its fractions after 48-hour incubation in contrast with cisplatin. Averaged value from three trials with triplicate readings. Different letters indicate significant differences (p < 0.05).

This study focused on the ability of *P. bundokalbo* spider venom to stimulate caspase 3/7 activity. Results in Figure 5 showed that untreated cells had a low relative luminescence unit (RLU) while high RLU values were observed in A549 cells treated with crude *P. bundokalbo* spider venom, venom fractions and cisplatin. Consistent with the cytotoxicity assay, fraction AT5-3 showed higher RLU value compared to the other fractions, which indicates caspase 3/7 activation. On the other hand, fractions AT5-5 and AT5-6 obtained RLU values similar to the untreated cells. 

### Phospholipase A_2_ Activity of *P. bundokalbo* Spider Venom

**Figure 6.**
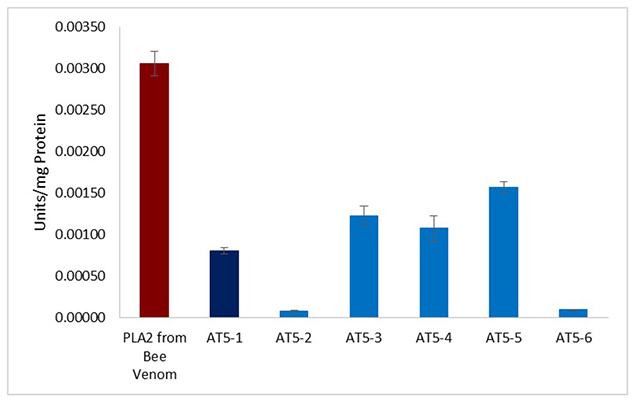
Presence of PLA_2_ activity of venom fractions from *P. bundokalbo*. Averaged value from three trials with triplicate readings. Different letters indicate significant differences (p < 0.05).

Phospholipase A_2_ is among the various types of enzymes present in spider venom. In this study, PLA_2_ activity of spider fractions was estimated using a colorimetric assay. Results (Figure 6) showed that fractions AT5-1, AT5-3, AT5-4, and AT5-5 had exhibited moderate PLA_2_ activity when compared with the standard PLA_2_ from bee venom. In contrast, fractions AT5-2 and AT5-6 presented minimal PLA_2_ activity.

## Discussion

*Phlogiellus bundokalbo* is one of the five known endemic tarantula species in the Philippines [12]. Though common in the country, there are still no known studies on the possible pharmacological application of its venom. Thus, the present study provides new insights on the medical relevance of the Philippine spider venom.

Most spider venoms consist of three main components, namely low-molecular-weight compounds, peptides (disulfide-containing neurotoxins and linear cytolytic peptides), and proteins (enzymes and neurotoxins), which have various biological functions [1]. Both inorganic and organic low-molecular-weight molecules have been found in spider venoms, including salts, carbohydrates, amino acids, biogenic amines, and acylpolyamines. Structural elucidation of the venom peptides from a Theraphosidae spider, *Phlogius* sp., using NMR and mass spectrometry, led to the discovery of an early eluting polyamine (PA_366_) consisting of a 2-hydroxy-3-(4-hydroxyohenyl) propanal aromatic head group that has a non-specific binding affinity to a range of proteins, as demonstrated by ProtoArray analysis, including zinc finger protein 501, and MAPK1 and MAP3K2 [8]. Hence, this finding also supports the participation of MAPK in the activation of apoptosis by the spider venom. 

In this research, we had partially separated components of *P. bundokalbo* spider venom using RP-HPLC. The chromatogram (Figure 1) revealed a highly distinct early eluting peak, namely AT5-1, which may correspond to acylpolyamines. Low-molecular-weight compounds like acylpolyamines usually elute during the early part of chromatographic separation due to their inherent polarity. Moreover, considering that PA_366_ was experimentally determined to be present in a wide range of geographically diverse spider species from the family Theraphosidae, it is likely that PA_366_ is also present in the venom of *P. bundokalbo*. Hence, it is necessary to purify and further analyze the venom fractions of *P. bundokalbo* to accurately determine the present compounds or peptides, and to link these substances to the innate bioactivity of the venom from the said spider.

Human lung adenocarcinoma cell line (A549) is commonly used in screening the bioactivity of various natural products. The A549 cell line is an adherent epithelial cell type that is sub-cultured easily under laboratory conditions, and is highly sensitive to treatment procedure. Furthermore, sub-culturing of A549 cells is under Biosafety Level 1 classification based on the U.S. Public Health Service Guidelines, making it more suitable for *in vitro* experimentation [13]. As mentioned previously, fraction AT5-3 exerted the highest inhibitory activity towards A549 cells. In this study, the cytotoxic activity of the spider venom and its fractions were compared to the activity of cisplatin. This drug is commonly used for the treatment of lung cancer, which involves binding with RNA, DNA and proteins to form adducts. Among these, DNA adducts are considered to be the key lesions facilitating the cytotoxic effect of cisplatin [14, 15]. However, exposure to cisplatin can also result in severe damage to normal post-mitotic tissues on account of oxidative stress, hence signifying a portion of oxidative stress in the dose-limiting toxicity of cisplatin [15]. 

Several studies had already shown the anticancer potential of spider venom. The crude spider venoms from *Haplopelma huwenum, H. hainanum,* and *M. raveni* triggered the expression of caspases in human neurogliocytoma (U251), human hepatocellular carcinoma (HepG2), and human myelogenous leukemia (K562) cell lines [11,13]. The activation of the initiator caspase, caspase-8, by the *M. raveni* venom in K562 promoted the cleavage and consequent activation of caspase-3, which is an effector caspase [11]. Concomitantly, this resulted in the cleavage of poly-ADP ribose polymerase (PARP) that initiated the execution of the apoptotic process. Similar results in HepG2 cells wherein activation of caspase-3 and -9, which led to cleavage of PARP by *H. hainanum* venom, was initiated by the depolarization of MMP and increased the cytochrome c level in the cytosol. This in turn caused the increased expression of Bax protein while Bcl-2 level was down-regulated, which indicated that the spider venom appears to stimulate apoptosis through the mitochondrial pathway [13].

Morphological features of apoptosis consist of retraction of pseudopods, decrease of cellular and nuclear volume, chromatin condensation, nuclear fragmentation, minor modifications of cytoplasmic organelles, plasma membrane blebbing, and phagocytosis [16]. On the other hand, necrosis is morphologically characterized by oncosis, swelling of organelles, plasma membrane rupture and subsequent loss of intracellular contents. After these cellular changes, the necrotic cells merge, and form a coarsely granular, amorphous, or hyaline material [17]. Nuclear condensation, which occurs during apoptosis, can be observed using Hoechst 33342, which is a cell-permeable DNA stain that specifically binds to adenine-thymine (A-T) regions of DNA. Hoechst 33342 binds in the minor groove of DNA, displays distinct fluorescence emission spectra, and is dependent on dye-base pair ratios. Apoptotic cells have condensed fragmented DNA, which is distinguishable from the condensed chromatin of normal mitotic cells [18]. To further discern the apoptotic cells, Annexin-V, Alexa Fluor^TM^ 488 conjugate dye was also used. This dye specifically binds to the phosphatidylserine (PS), which is translocated to the plasma membrane during apoptosis. Binding of Annexin V to membranes that contain PS occurs in a cooperative manner with respect to calcium ion concentration [19].

Caspase activation is also among the previously described characteristics of spider venom, and is also related to apoptosis. While there are some fractions of *P. bundokalbo* spider venom that can induce caspase 3/7 activation, there are also components that are incapable of such induction. As mentioned previously, fraction AT5-3 had the highest RLU values among the fractions of *P. bundokalbo*, which is in line with its cytotoxic activity towards A549 cells. On the other hand, AT5-5 had the least inhibitory activity towards A549 cells while AT5-6 was able to cause cell lysis at high concentrations. Furthermore, no PS fluorescent signals were detected based on the cytological characterization of A549 cells treated with AT5-5 and AT5-6. In this context, it can be inferred that the fraction AT5-5 does not exert either cytotoxic or apoptotic effects towards A549 cells. The observed leaking of cellular components caused by the loss of cell membrane integrity may be attributable to AT5-6 possibly having induced necrotic cell death. As presented in a previous study, flow cytometric analysis of TE13 and HeLa cells treated with crude *M. raveni* spider venom revealed that the main mode of cell death was necrosis, with minimal apoptotic cells. The researchers eventually concluded that apoptosis, necrosis, and cell lysis of tumors are the possible mechanisms by which the crude venom of *M. raveni* inhibited tumor growth [20]. It is therefore important to identify the responsible spider venom component and to determine the proper concentration in order to elicit the desired mode of cancer cell death.

Stimulation of apoptosis by spider venoms also includes the activation of mitogen-activated protein kinase (MAPK). The MAPK genes are associated with different types of cancer, which when activated can affect cell proliferation, invasion and metastasis [8]. As depicted in a previous study, inhibition of p38 MAPK using SB20350 contributed to a significant decrease in caspase 3 activity in A549, thereby decreasing the cytotoxicity of spider venom [10]. These interesting insights about the interaction of spider venom components with MAPK could lead to a deeper understanding of the underlying mechanism that is involved in the apoptotic response of the spider venom. The mechanism of most anticancer drugs is closely correlated with the stimulation of MAPK JNK and p38 that are activated in response to various intrinsic and extrinsic stresses. In particular, p38 activation induces apoptosis by phosphorylating or indirectly down-regulating pro-survival Bcl-2 family proteins under conditions such as cellular stress including ROS [21].

On the other hand, there are studies demonstrating the interplay of ERK activation in survival and death of cancer cells. ERK is activated by mitogenic stimuli, such as growth factors and cytokines. Constitutive activation and overexpression of ERK are frequently observed in many cancer cells. Nevertheless, accumulating evidence revealed that ERK activation could take effect in cancer treatment by inducing apoptosis, autophagy and senescence. This was demonstrated in a recent study that reported an apoptotic effect of BPIQ against NSCLC. The authors discovered that at a high BPIQ concentration, ERK activation is partially responsible for its antiproliferative activity whereas at a sub-lethal dose of BPIQ, inhibition of ERK occurred that promoted the attenuation of NSCLC migration [21].

The presence of venom peptides could also contribute to cytotoxicity towards cancer cells. Animal venoms with an inhibitory effect on the release or activity of matrix metalloproteinases (MMP) are capable of reducing cancer cell motility, tumor cell invasion, and metastatic potential of malignant tumors [5]. Aside from the proteolytic activity of MMPs, cancer cell proliferation, migration and invasion are also affected by ionic channel activity [22, 23]. For instance, inhibition of voltage-gated sodium channels by tetrodotoxin reduced *in vitro* invasion of NSCLC cell lines H23, H460 and Calu-1 by 40-50% [24]. Spider venom may inhibit cancer cell growth and proliferation, affect cellular migration, induce apoptosis or cause cell cycle arrest by binding to specific targets on cell membrane, thus blocking specific ion channel, inhibiting angiogenesis and other mechanisms [13, 25]. These resulting activities might be due to synergistic interactions between the different venom compound groups, such as ions, low-molecular-weight compounds, enzymes, neurotoxins, low-molecular-weight cationic peptides, and α-helical cationic peptides [26]. There are some cationic linear antimicrobial peptides from spider venom - such as cecropins and magainins - that act more selectively towards cancer cells due to the elevated negative surface charge and higher transmembrane potential level of cancer cells compared to normal cells [24]. In this context, determining the active components of the spider venom that are responsible for the inhibitory activity towards cancer cells is crucial in classifying the selectivity of the spider venom towards cancer cells. Decreasing the molecular hydrophobicity potential of the N-terminal part of Ltc2a by introduction of polar residues or deletion of three N-terminal residues would help in eliminating the unwanted hemolytic and cytotoxic activity of the venom peptide towards normal cells [27].

Spider venom also contains PLA_2_, which hydrolyzes the fatty acid from the membrane phospholipids that subsequently release eicosanoids and other related bioactive lipid mediators [28]. The presence of PLA_2_ activity in some fractions of *P. bundokalbo* spider venom - such as AT5-1 and AT5-3 - may promote cytotoxicity towards A549 cells. The possible involvement of PLA_2_ in the cytotoxicity of the spider venom may be associated with its ability to recognize and attach to phosphatidylserine [29]. Externalization of phosphatidylserine in cells is a marker of apoptosis; therefore, hemolysis of A549 cells exposed to *P. bundokalbo* spider venom may promote the production of inflammatory mediators. However, cancer cell cytotoxicity is not always attributable to the presence of PLA_2_. 

In the case of fraction AT5-6, where it has a high toxicity towards A549 as seen in MTT assay result, PLA_2_ activity was not observed. This is probably due to the specific interaction of PLA_2_ constituents towards lipid membranes. PLA_2_ with tryptophan on the lipid-binding surface displays the highest activity toward neutral lipid substrates, whereas PLA_2_ with an excess of basic residues on the lipid-binding surface interacts mainly with negatively charged surfaces [30]. PLA_2_ is commonly found in snake venoms; there are reports suggesting the presence of this enzyme in the venom of some species of spiders [1, 31, 32]. The crude venom from *Phoneutria boliviensis* and its two RP-HPLC fractions (eluted at 33% and 37% acetonitrile) showed PLA_2_ activity by completely hydrolyzing 4-nitro-3-octanoyloxy-benzoic acid [30]. Spider venom extracted from *Hippasa partita, Hippasa agelenoides,* and *Hippasa lycosina* demonstrated various enzymatic activities such as proteolysis, hydrolysis of hyaluronic acid, and PLA_2_ activity. A similar study associated the cytotoxicity of *H. partita* venom towards Ehrlich ascites tumor cells with the presence of PLA_2_ and sphingomyelinase D [31]. Although only the protein components of the venom was determined in the prior / present study, the possible influence of other non-enzymatic components of the spider venom that are capable of inducing cytotoxicity should not be ruled out. 

## Conclusion

In summary, this research has shown the potential cytotoxic activity of *Phlogiellus bundokalbo* spider venom from Mindanao, Philippines, against human lung adenocarcinoma (A549) cells, which is also comparable to the activity of the standard drug cisplatin. By inducing caspase 3/7 activity, the venom fractions present promising inhibitory activity that could induce either apoptotic or necrotic cell death, depending on the fraction type and its concentration.

Therefore, an *in vitro* cytotoxicity assay on human lung adenocarcinoma has proposed that the venom of *P. bundokalbo* facilitates cytotoxicity and functions as a potential anticancer agent. Moreover, elaboration of findings on the structural aspects responsible for this activity will be pivotal in considering further development as a clinically useful pharmaceutical agent.
